# Murine Anorectic Response to Deoxynivalenol (Vomitoxin) Is Sex-Dependent

**DOI:** 10.3390/toxins7082845

**Published:** 2015-07-29

**Authors:** Erica S. Clark, Brenna M. Flannery, James J. Pestka

**Affiliations:** 1 Department of Food Science and Human Nutrition, Michigan State University, East Lansing, MI 48824, USA; E-Mails: clarke27@msu.edu (E.S.C.); flanne12@msu.edu (B.M.F.); 2 Center for Integrative Toxicology, Michigan State University, East Lansing, MI 48824, USA; 3 Department of Microbiology and Molecular Genetics, Michigan State University, East Lansing, MI 48824, USA

**Keywords:** trichothecene, mycotoxin, anorexia, weight loss, IL-6, mouse, sex dependence

## Abstract

Deoxynivalenol (DON, vomitoxin), a common trichothecene mycotoxin found in cereal foods, dysregulates immune function and maintenance of energy balance. The purpose of this study was to determine if sex differences are similarly evident in DON’s anorectic responses in mice. A bioassay for feed refusal, previously developed by our lab, was used to compare acute i.p. exposures of 1 and 5 mg/kg bw DON in C57BL6 mice. Greater anorectic responses were seen in male than female mice. Male mice had higher organ and plasma concentrations of DON upon acute exposure than their female counterparts. A significant increase in IL-6 plasma levels was also observed in males while cholecystokinin response was higher in females. When effects of sex on food intake and body weight changes were compared after subchronic dietary exposure to 1, 2.5, and 10 ppm DON, males were found again to be more sensitive. Demonstration of male predilection to DON-induced changes in food intake and weight gain might an important consideration in future risk assessment of DON and other trichothecenes.

## 1. Introduction

Deoxynivalenol (DON, vomitoxin) is a trichothecene mycotoxin produced by the fungus *Fusarium graminearum* that contaminates corn, wheat, and barley [1]. DON is highly resistant to heat processing and can enter human and animal food. A recent study reported high levels of DON contamination in corn (76%) and wheat (79%) samples obtained from North America, suggesting that exposure to this mycotoxin is frequent [2]. In experimental animals, adverse effects of acute exposure include symptoms of gastrointestinal illness while chronic exposure can lead to growth retardation and immunotoxic effects [3,4,5,6]. In species capable of emesis (e.g. pig, mink), DON rapidly induces vomiting [7,8]. Rodents, however, are incapable of vomiting and instead exhibit feed refusal following exposure to this toxin [9,10].

Previous investigations suggest that male animals are more sensitive than female animals to the adverse effects from DON consumption [11,12,13,14]. Studies in swine have reported greater sensitivities in males to weight suppression and food intake than females [12,15]. An investigation conducted in ICR mice found that males fed DON containing diets showed greater growth depression and lower food intake than female mice [13]. The same study also reported that the amount of DON consumed by body weight was lower in male mice (1.49 mg DON/kg bw/day) than in female mice (1.59 mg DON/kg bw/day) further supporting the contention that the response to this toxin differs by sex. A two-year feeding study in B6C3F1 mice found that males fed DON diets consumed less food than females, though this did not lead to a significant reduction in body weight [4]. Finally, previous investigations by our laboratory identifying a male predilection to DON induced IgA nephropathy also reported greater weight suppression in male mice than female mice [11,14,16].

DON-induced feed refusal have been linked to the induction of proinflammatory cytokines (*i.e.*, IL-1β, IL-6, and TNF-α), which are known to have anorectic actions in animals and humans [17,18,19]. DON exposure has previously been shown to induce these proinflammatory cytokine expressions in the female mouse model [20,21]. Since immune dysregulation of IgA is greater in male mice than female mice, comparison of sex differences in these cytokines is also of interest.

Elevation of the satiety hormones cholecystokinin (CCK) and peptide YY (PYY) have also been observed upon DON exposure [22,23]. CCK is a hormone that is secreted by I cells within the small intestine and increases the expression of anorexigenic peptides including cocaine and amphetamine regulated transcript (CART) [24]. PYY is a 36-amino acid peptide that also decreases food intake by increasing the expression of anorexigenic peptides and is secreted by the L cells of the colon and ileum [25]. While previous studies have explored elevation of these hormones in female animals following DON exposure, the effect of DON on satiety hormones in males has yet to be addressed.

The aims of this study were to evaluate and characterize murine sex differences in food refusal response after acute and dietary DON exposure. Study 1 addressed sex differences in feed refusal response to acute i.p. DON exposure. Study 2 compared DON tissue concentrations, as well as proinflammatory cytokine and satiety hormone responses to acute DON exposure in males and females. The objective of Study 3 was to compare sex differences in food intake and body weight suppression upon exposure to dietary DON. The results indicate that male mice were more sensitive than female mice to acute i.p. and dietary DON exposure and that these differences correspond to slower toxin organ clearance and an increased IL-6 response with acute i.p. exposure.

## 2. Results and Discussion

### 2.1. Study 1

#### Feed Refusal upon Acute DON Exposure Is Greater in Males than Females

**Figure 1:**
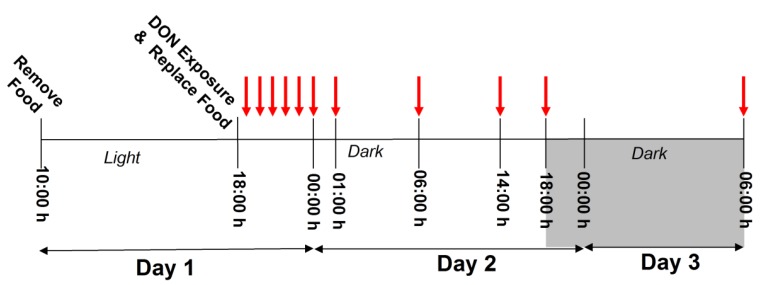
Study 1 experimental design. On Day 1, mice were fasted from 10:00 a.m. to 6:00 p.m. then exposed to DON treatments or vehicle control. Food was immediately replaced following exposure and food measurements were recorded hourly from 1 to 7 h PI, and at 12, 20, 24, and 36 h PI as indicated by arrows.

**Figure 2:**
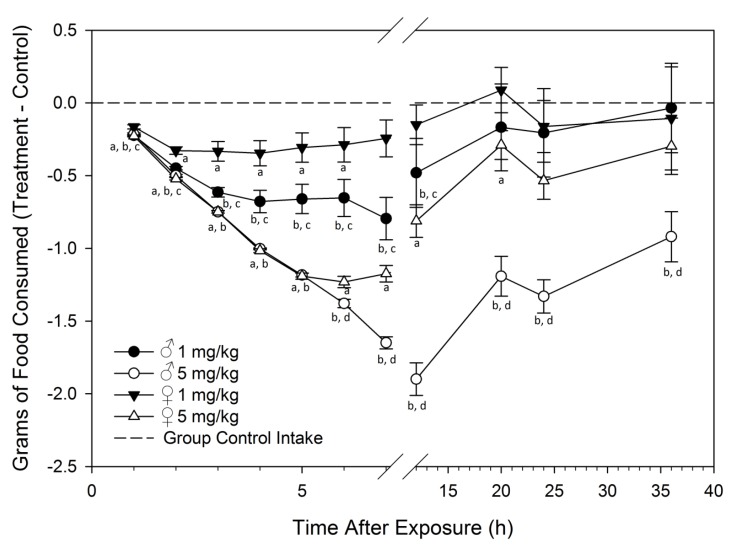
Male mice are more sensitive to DON-induced feed refusal than females following acute i.p. exposure. Food intake is cumulative at each time point and was normalized to group control average. Data are mean ± SEM (*n* = 10/gp). Statistically significance differences are indicated as follows: a = DON-dosed female different from female control; b = DON-dosed male different from male control; c = male different from female at 1 mg/kg bw DON; and d = male different from female at 5 mg/kg bw DON (*p* < 0.05).

The effects of acute i.p. exposure to DON on food intake were compared in male and female mice over a period of 36 h (Figure 1). Male mice treated with 1 mg/kg bw DON ate 45% less food then control males at 6 h post-injection (PI) (Figure 2). After 6 h, male mice showed recovery with cumulative food intake nearly reaching that of controls 36 h PI. In contrast, female mice treated with 1 mg/kg bw DON ate 24% less food than female control mice at 6 h PI and began to show recovery in food intake at 5 h PI. Male mice treated with 5 mg/kg bw exhibited the greatest difference in food intake from the control males between 7 and 12 h PI, consuming 7% of food intake compared to control males. Food intake in males exposed to 5 mg/kg bw DON started to increase after 12 h PI, however, at 36 h PI males had consumed only 82% of what control males had eaten. Conversely, female mice treated with 5 mg/kg bw DON started to show recovery in food intake at 7 h after DON treatment and had eaten 33% of that by control females. At the final time point of 36 h PI, food intake of female mice treated with 5 mg/kg bw DON had almost completely recovered with the animals having cumulatively consumed 95% of the food intake of control females.

While this is the first comparison of murine sex differences in DON-induced anorectic effects, acute feed refusal has been previously described in B6C3F1 female mice. Flannery *et al.* [10,22] observed that by 4 h food intake had recovered in female B6C3F1 mice treated with 1 mg/kg bw DON i.p. In this study, we found that C57BL6 females at the same time point and dose continued to show feed refusal, consuming 37% food than control females. These findings are consistent with reports of greater sensitivity of C57BL6 females to body weight reduction and mortality with DON exposure in comparison to B6C3F1 females [11].

### 2.2. Study 2

#### 2.2.1. DON Organ Concentrations Are Higher in Male Mice after Acute DON Exposure

Tissues were analyzed for DON following acute exposure to 1 mg/kg bw at 1, 2, and 4 h PI. DON concentrations were represented as DON equivalents, as the ELISA used to quantify DON is 100% cross-reactive with the DON metabolite deoxynivalenol-3-glucuronide (DONGlcA) (Supplementary Data, Figure S1). At 1 h PI, kidney, liver, and heart DON equivalent concentrations were higher in male mice than female mice (Table 1). Males had the highest concentration in kidney followed by the plasma. Female mice at this time point had the highest concentration of DON equivalents in the plasma, followed by the kidney. The rank order of DON equivalent concentrations in all other organs for males and females was: liver > heart > spleen > brain. Similar tissue distribution patterns have previously been reported in B6C3F1 mice and pigs [26,27]. High concentrations of DON equivalents in the kidney of male mice at 1 h after DON treatment could indicate that male mice are excreting the toxin more slowly than female mice.

No differences in DON equivalent organ concentrations between males and females were observed at 2 h PI (data not shown). However, DON equivalent concentrations were significantly higher in males than females in all organs and plasma at 4 h post exposure, with the exception of the brain (Table 1). Interestingly, the brain showed the lowest change in DON equivalents from 1 to 4 h, with the concentration remaining to be approximately 50% of the 1 h levels measured in both male and female mice while all organs had decreased DON levels from 7% to 14% of the 1 h measurement. Higher toxin levels remaining in the tissues of male mice could be a contributing factor to the increased feed refusal seen in males.

**Table 1:** DON equivalent concentrations are higher in male mice at 1 and 4 h post acute i.p. exposure to 1 mg/kg bw DON. DON equivalent concentrations are nmol/g. DON is reported as DON equivalents as the ELISA was found to be completely cross reactive with DON3GlcA. Control animals did not have detectable levels of DON (data not shown). Data are mean ± SEM (*n* = 5–6/gp).

Organ	DON Equivalents (nmol/g)			
	1 h		4 h	
	♂	♀	♂	♀
Kidney	3.85 ± 0.3 *	2.21 ± 0.2	0.30 ± 0.03 *	0.17 ± 0.01
Liver	2.60 ± 0.2 *^p^* ^= 0.09^	1.86 ± 0.3	0.36 ± 0.1 *	0.14 ± 0.02
Plasma	2.72 ± 0.2	2.33 ± 0.2	0.20 ± 0.01 *	0.12 ± 0.01
Heart	2.24 ± 0.1 *	1.60 ± 0.2	0.19 ± 0.01 *	0.11 ± 0.01
Spleen	1.44 ± 0.1	1.16 ± 0.2	0.17 ± 0.01 *	0.10 ± 0.01
Brain	0.61 ± 0.01	0.64 ± 0.04	0.33 ± 0.02	0.30 ± 0.05

* indicates significantly different from female at specified time point (*p* < 0.05).

Overall, these data suggest that males may differ from females in absorption and/or clearance of DON. One possible metabolic difference is that males have a reduced capacity to glucuronidate, thus excrete the toxin. Sex differences have previously been identified in mRNA levels of UDP-glucuronosyltransferases (UGTs), the family of enzymes responsible for DON glucuronidation [28,29]. Analyzing possible sex differences in DON metabolism will be an important objective for future studies.

#### 2.2.2. Plasma IL-6 Is Higher in Male Mice than Female Mice upon Acute DON Exposure

**Figure 3:**
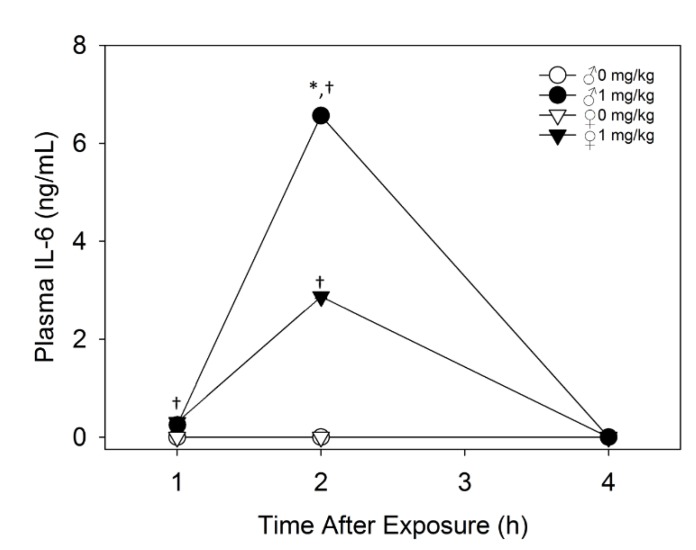
Male mice exhibit higher IL-6 plasma levels at 2 h post-acute i.p. exposure to 1 mg/kg bw DON. Data are mean ± SEM (*n* = 4/rep). Asterisk indicates statistical significance from female at time point and dagger indicates significance from group control at time point (*p* < 0.05).

When the effects of acute exposure to 1 mg/kg bw DON on plasma levels of proinflammatory cytokines were determined, DON treated males and females both had significantly higher IL-6 plasma levels than their respective group control at 1 and 2 h PI (Figure 3). Plasma IL-6 concentrations were elevated 2.25 fold higher at 2 h after DON treatment in male mice when compared to female mice. The high increase in this anorectic cytokine could be another factor to male sensitivity to acute DON-induced anorexia.

Plasma TNF-α and IL-1β were not induced by DON (data not shown). These findings are consistent with previous studies reporting very low levels of IL-1β and no increase in TNF-α plasma levels with treatments below 12.5 mg/kg bw DON [30,31].

**Figure 4:**
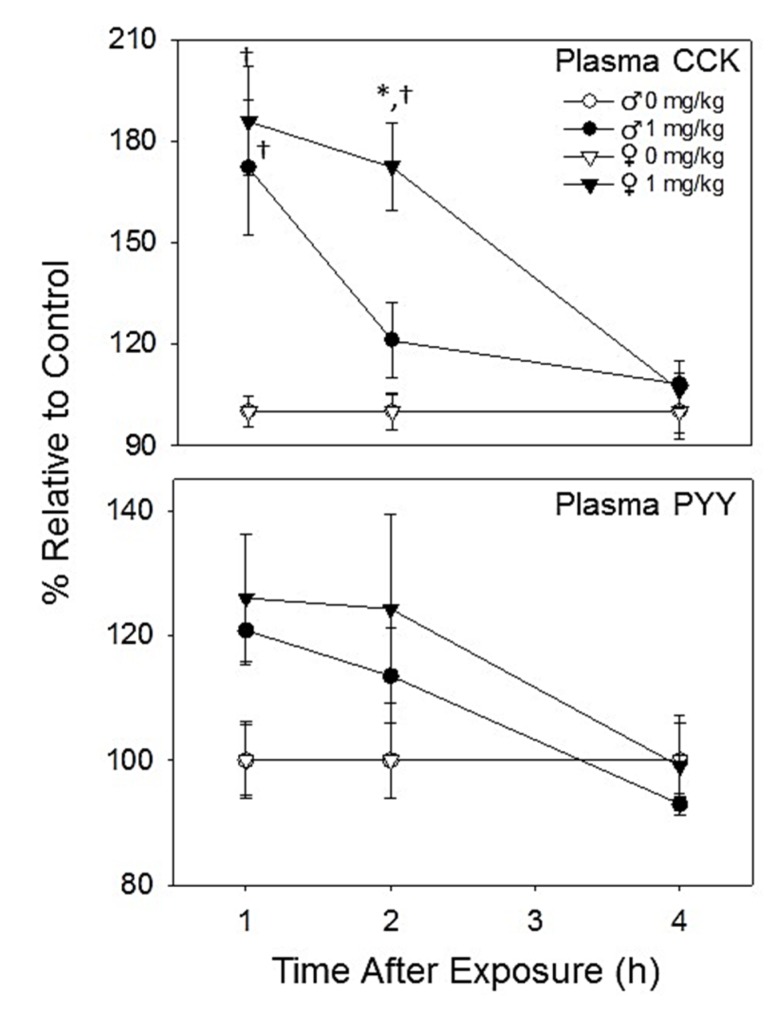
DON induces plasma CCK and PYY elevation. All values are reported as percent of the group control as control females had higher baseline levels of both gut satiety hormones. Data are mean ± SEM (*n* = 5–6/gp). Asterisk indicates statistical significance from male at time point and dagger indicates significance from group control at time point (*p* < 0.05).

#### 2.2.3. Plasma CCK and PYY Are Increased in Both Female and Male Mice with DON Exposure

DON treatment increased plasma CCK and PYY concentrations in both male and female mice in comparison to control animals at 1 and 2 h PI, returning to baseline levels by 4 h PI (Figure 4). Relative levels of gut satiety hormones in DON-treated female mice tended to be slightly higher than male mice, but only plasma CCK at 2 h being significantly higher in females. Thus, increased sensitivity of male mice to DON-induced feed refusal could not be explained by differences in either of these two satiety hormones.

### 2.3. Study 3

#### 2.3.1. Initial Food Intake Is Suppressed in Male Mice But Not Females Exposed to Dietary DON

When food intake was measured during Study 3, DON-fed male mice exhibited significant differences in the amount of food consumed at 1 and 2 days (Table 2). At these two time points there was significant negative correlation between decreasing food consumption with increasing DON treatments. At 2 days of treatment, males on diets containing 2.5 and 10 ppm DON were eating 15% and 25% less respectively than control animals. Translating such decrease to a 2000 kcal diet would correspond in reduction of eating 1700 and 1500 calories, respectively. In comparison, the females did not display a significant suppression in food intake. While female mice showed a trend of decreasing food consumption on day 1 of treatment, the correlation was only approaching statistical significance. After 2 days, mice fed diets containing DON progressively began to shred the food pellets precluding accurate food recovery after this time point.

**Table 2:** Food consumption is decreased in male mice fed DON containing diets. Values are percent of group control food intake. Data are mean ± SEM (*n* = 6/group).

Group	% Control Food Intake 0–24 h	% Control Food Intake 24–48 h
♂ **1 ppm**	99.5 ± 3.8 *	95.2 ± 7.2 *
♂ **2.5 ppm**	85.7 ± 7.3 *	85.0 ± 4.9 *
♂ **10 ppm**	56.9 ± 10.2 *	74.9 ± 5.6 *
♀ **1 ppm**	100.7 ± 4.2	95.6 ± 4.6
♀ **2.5 ppm**	99.5 ± 8.1	101.4 ± 5.9
♀ **10 ppm**	84.0 ± 7.8	92.3 ± 8.0

* indicates statistical significance in correlations between increasing dose and decreasing food intake at time point (*p* < 0.05). Correlation coefficients were: (males 0–24 h; *r* = −0.731; *p* = 0.00009), (females 0–24 h; *r* = −0.384, *p* = 0.06), (males 24–48 h; *r* = −0.495; *p* = 0.01), and (females 24–48 h; *r* = −0.16; *p* = 0.46).

The greater suppression of food intake with dietary DON exposure in male mice compared to females was consistent with the observation of an increased anorectic effect in males *versus* females in our acute i.p. DON exposure Study 1. These results also correspond to sex differences in food intake with DON exposure that have previously been reported [4,13,15]. Studies in ICR and B6C3F1 mice reported a greater decrease in male food intake with dietary DON exposure when compared to females [4,13]. A meta-analysis performed in swine research also reported that food intake with DON exposure was suppressed in males compared females, with reductions of 20% and 3%, respectively [15].

#### 2.3.2. Dietary DON Exposure Causes Greater Suppression of Weight Gain in Male Mice

**Figure 5:**
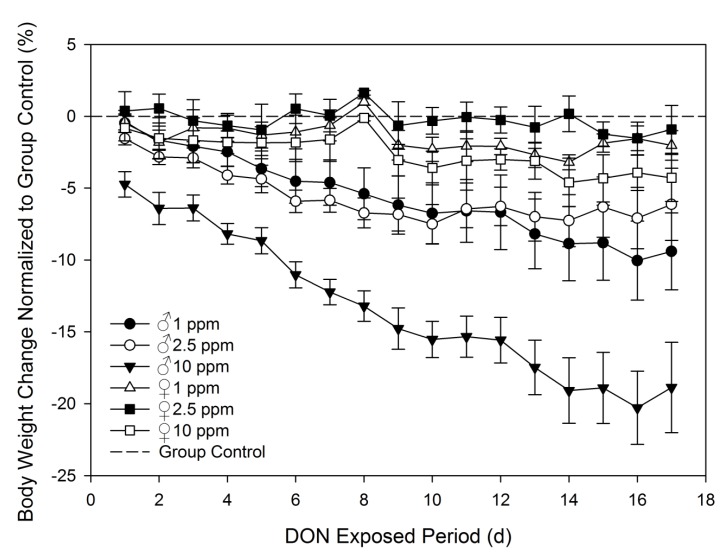
Male mice are more sensitive to DON-induced anorexia than female mice upon dietary DON exposure. Changes in daily body weights were determined from weight at study beginning and normalized to group control weight gain. Data are mean ± SEM (*n* = 6/group). Statistical significance is indicated in Table 2.

When the effect of dietary exposure to DON at 0, 1, 2.5, and 10 ppm on body weight were compared over 17 days, male mice showed a significant decrease in body weight while female mice did not (Figure 5; Table 3). Males fed 10 ppm DON exhibited significantly suppressed weight gain in comparison to control males and females fed 10 ppm DON diet beginning at day 3 of treatment and maintained this suppression until the end of the experiment. At the termination of the study, males fed with 10 ppm DON diet weighed 17% less than group controls. After 17 days of exposure to the diet containing 10 ppm DON, female mice exhibited only a 6% depression of body weight gain compared to group controls. Males on diets containing 1 and 2.5 ppm DON weighed 7% less than group at the end of the study, while females on these diets weighed only weighed 2% less than group controls. Adaptation to the toxin and increased metabolic capacity of the toxin are possible explanations for weight loss leveling near the end of the study.

The results presented here are consistent with previous animal studies examining sex differences with dietary DON exposure [4,11,12,13,15]. In a study conducted by Cote *et al.* [12] in swine, researchers found that castrated male swine (barrows) on diets containing 3.1 ppm and 5.8 ppm DON had increased difficulty with consistent weight gain in comparison with female swine. That study also reported that male swine did not resume normal growth rates after being removed from treatment diets, while female swine growth rates recovered. A meta-analysis found an average of weight reduction of 34% in DON treated barrows, while female weights were only reduced by 2% [15]. Rotter *et al.* [13] found that male ICR mice on diets containing 2–8 ppm DON showed a greater suppression of weight gain than females over a 14 days period compared to control animals. They also reported that animals appeared to show adaptation to the DON diets in the second week of exposure. A two-year feeding study conducted by Iverson *et al.* [4] examined sex differences to prolonged DON exposure in B6C3F1 mice. While they did not observe greater weight suppression in male mice compared to female mice, the study did report that males fed diets containing 5 and 10 ppm DON did consume significantly less than control males and no significant differences in food consumption were reported in female mice.

**Table 3:** Statistical analysis of Figure 5 data. Significant differences are indicated as follows: a = different from group control body weight percent change and b = different from female body weight percent change at same dose and day (*p* < 0.05). No statistical significance was observed in females treated with DON in comparison to group control.

Treatment	Day								
	0	1	2	3	4	5	6	7	8
♂ 1 ppm	-	-	-	-	-	-	-	-	a, b
♂ 2.5 ppm	-	-	-	-	-	-	-	-	a, b
♂ 10 ppm	-	-	-	a, b	a, b	a, b	a, b	a, b	a, b
	9	10	11	12	13	14	15	16	17
♂ 1 ppm	a	a, b	-	a, b	a	-	-	a, b	a
♂ 2.5 ppm	a, b	a, b	-	a, b	-	-	-	-	-
♂ 10 ppm	a, b	a, b	a, b	a, b	a, b	a, b	a, b	a, b	a, b

#### 2.3.3. Dietary DON Exposure Contributes to Elevated Liver DON Equivalents in Male Mice

**Figure 6:**
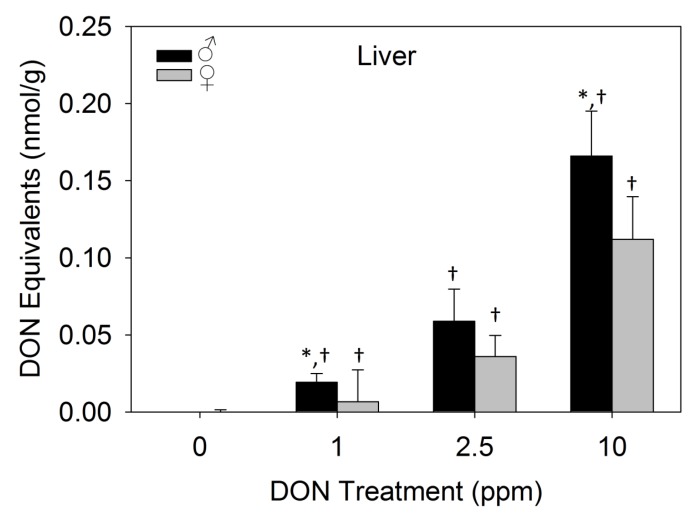
Male mice have higher liver DON equivalents than females after dietary toxin exposure. Data are mean ± SEM (*n* = 6/gp). Asterisk indicates statistical significance from female on same treatment diet and dagger indicates significance from preceding treatment dose within sex (*p* < 0.05).

DON equivalents in the organs and plasma after 17 days of dietary DON exposure were compared in male and female mice. Liver DON equivalents in male mice fed 1 and 10 ppm DON diets were statistically significantly higher than females by 2.9 and 1.5 fold, respectively (Figure 6). DON equivalents in the livers of males fed 2.5 ppm DON diets were 1.6 fold higher than the concentrations found females, though this difference was not statistically significant. Liver DON equivalents significantly increased with diets containing higher amounts of DON in both male and female mice. Sex differences in toxin concentrations in all other organs analyzed were not statistically significant, with the exception of kidney DON equivalent concentrations being higher in females than males fed 1 ppm DON diets (Supplementary Data, Figure S2). As animals were allowed *ad libitum* access to food prior to euthanasia, the amount of toxin consumed and time of consumption are unknown. While unrestricted access to feed prior to euthanasia complicates data interpretation, identifying higher levels of DON equivalents in male mouse livers in both the acute i.p. exposure Study 1 and Study 3 could also suggest that male mice could absorb more toxin and/or require more time to metabolize the toxin.

#### 2.3.4. Proinflammatory Plasma Cytokines Were not Detectable after 17 days of Dietary DON Exposure

Plasma levels of the proinflammatory cytokines IL-6, IL-1β, and TNF-α were not detectable in either male or female mice after 17 days of dietary DON exposure at any treatment level. These findings are consistent with a previous study also reporting no detection of plasma levels of IL-6, IL-1β, or TNF-α in mice exposed to 2 mg/mL DON in drinking water for 36 days [32].

## 3. Experimental Section

### 3.1. Animals

The Institutional Animal Care and Use Committee at Michigan State University approved all animal experiments. Adult male and adult female C57BL6 mice (12 weeks) were purchased from Charles River Laboratories (Portage, MI, USA). Mice were housed singly in polycarbonate cages with sifted aspen bedding on a 12 h light/dark cycle, with constant temperature (21–24 °C) and humidity (40%–55%). In all experiments, mice were fed high fat pellet diet (45% kcal from fat; Research Diets, Inc., New Brunswick, NJ, USA) 1 week prior to DON exposure. High fat diet was used as it was previously determined to provide the most efficient food recovery [10].

### 3.2. DON

DON used for i.p*.* injections and DON amended diet was obtained from Dr. Tony Durst (University of Ottawa, Canada) and purity was verified to be 98% by elemental analysis (Galbraith Labs, Knoxville, TN, USA). For all i.p. injections, DON was appropriately dissolved in Dulbecco’s phosphate buffered saline (PBS; Sigma-Aldrich, St. Louis, MO, USA) to yield 100 µL injection volumes. High fat pellet diets containing 0, 1, 2.5 and 10 ppm DON were formulated by Research Diets, Inc. DON concentration of diets were confirmed using the Veratox high sensitivity (HS) enzyme-linked immunosorbent assay according to manufacturer protocol (ELISA; Neogen, Lansing, MI, USA).

### 3.3. Experimental Design

#### 3.3.1. Study 1

The effects of sex on food intake after acute i.p. DON exposure were assessed, as illustrated in Figure 1. Upon arrival, mice were acclimated to food and handling according to our previously described feed refusal assay [10]. After acclimation, male and female mice (*n* = 10/group) were fasted from 10:00 a.m. to 6:00 p.m., and i.p. injected with either 0 (PBS vehicle control), 1 or 5 mg/kg bw DON (Figure 1). Food consumption was measured hourly 1 to 7 h post exposure, and at 12, 20, 24 and 36 h post exposure. Measurements were conducted under red light conditions during dark cycle.

#### 3.3.2. Study 2

The effects of sex on tissue DON equivalent concentrations and plasma concentrations of proinflammatory cytokines and satiety hormones were measured after acute i.p exposure to the toxin. Male and female mice (*n* = 5–6/group), were similarly fasted as in Study 1 and exposed to 1 mg/kg bw DON in PBS or PBS vehicle via i.p. injection. Mice were euthanized via CO_2_ chamber at 1, 2, and 4 h post exposure without food replacement. Blood was collected via cardiac puncture and the kidney, liver, spleen, heart and brain were collected and immediately snap frozen. Plasma was isolated from blood by centrifugation at 3500× *g* for 10 minutes at 4 °C. Plasma and organs were stored at −80 °C until analysis.

#### 3.3.3. Study 3

The effects of sex on response to dietary DON exposure were assessed. After acclimation, male and female were randomized into equal weight groups by sex (*n*= 6/group) and placed on high fat diets containing 0, 1, 2.5 and 10 ppm DON. Body weights were measured daily at 10:00 am for 17 days. The study was terminated at 17 days as body weight changes appeared to be stabilizing at this point. Food intake measurements were attempted the entire 17 days period. However, after 2 days, mice on diets containing DON progressively began to shred the pellets into fine particles that were unrecoverable from sieved bedding and precluded accurate food recovery. After 17 days of DON exposure, mice were euthanized via CO_2_ chamber at 8:00 a.m. Food access to treatment diets was *ad libitum* prior to euthanasia. Blood was collected via cardiac puncture and the kidney, liver, spleen, heart and brain were collected and immediately snap frozen. Plasma was isolated from blood. Plasma and organs were stored at −80 °C until analysis.

### 3.4. Analytical

#### 3.4.1. DON Quantification

DON quantification in plasma and organs were analyzed using the Veratox high sensitivity (HS) ELISA (Neogen, Lansing, MI, USA) as described previously with slight modifications [26]. Briefly, organs were homogenized 1:1 in PBS (except the heart, which was homogenized 1:2). Tissue homogenates were heated at 100 °C for 5 min and then centrifuged at 14,000 *g* for 10 min at 4 °C. The resulting supernatant was analyzed for DON using a F3 ELISA plate reader at 650 nm and Softmax software (Molecular Devices, Menlo Park, CA, USA). DON is reported as DON equivalents as the ELISA was found to be completely cross-reactive with DON3GlcA obtained from Dr. Philipp Fruhmann (Vienna University of Technology, Austria) (Supplementary Data, Figure S1). DONGlcA-3 is the major metabolite formed in rat liver. Cross reactivity with other potential glucuronides was not determined. Since we did not measure ratio of active *vs*. conjugated DON, results are reported as DON equivalents.

#### 3.4.2. Proinflammatory Cytokine Analyses

Plasma levels of the proinflammatory cytokines IL-6, IL-1β, and TNF-α were determined using Duoset ELISAs from R&D Systems (Minneapolis, MN, USA). Equal volumes of mouse plasma samples (*n* = 5–6/group Study 2; *n* = 6/group Study 3) were pooled within groups and ran in technical replicates (*n* = 4 rep) because volumes of individual samples were low.

#### 3.4.3. Satiety Hormone Analyses

Plasma levels of the gut satiety hormones CCK and PYY_3–36_ in with plasma samples from Study 2 (*n* = 6/group) were analyzed using ELISA kits for CCK (CCK_26–33_, nonsulfated; human-, rat-, and mouse-specific) and PYY (PYY_3–36_; mouse-, rat-, porcine-, and canine-specific) (Phoenix Pharmaceuticals, Burlingame, CA, USA). CCK and PYY were not measured in Study 3 as animals had *ad libitum* access to food prior to study termination.

### 3.5. Statistical Analysis

Statistical analysis was conducted by SigmaPlot version 11.0 (Jandel Scientific; San Rafael, CA, USA, 2009). Statistical comparisons between sexes made at each time point using a Student’s t-test, unless normality failed. If normality was not met, a Mann–Whitney Rank Sum test was performed. Statistical comparisons between sex and dose were made at each time point using a one-way analysis of variance (ANOVA), unless normality failed. A Kruskal–Wallis one-way analysis of variance by ranks was performed if normality was not met. Student–Newman–Keuls was used in all *post-hoc* analysis for parametric and non-parametric animal groups of equal numbers. Dunn’s *post-hoc* analysis was used in non-parametric analysis of animal groups with unequal numbers. Pearson product movement correlations were performed to determine statistical significance between food consumption and treatment levels of DON diets by sex and day in Study 3. Differences were considered significant when *p* < 0.05.

## 4. Conclusions

The results presented in this study indicated that male mice were more sensitive than females to anorexia induction after acute and dietary DON exposure. These effects corresponded to decreasing body weight in males fed DON containing diets. As toxin organ clearance was slower in male mice than female mice, future studies should evaluate possible sex differences in DON uptake, metabolism and excretion. Relating sex differences to increased vulnerability to DON will be an important consideration in future risk assessment of this and other trichothecenes.

## Supplementary Materials

Supplementary materials can be accessed at: http://www.mdpi.com/2072-6651/7/8/2845/s1.
