# Supplementary material for: Murine Anorectic Response to Deoxynivalenol (Vomitoxin) Is Sex-Dependent
Source: Toxins (Basel). 2015 Jul 29;7(8):2845–59. doi: 10.3390/toxins7082845 (PMC4549728; doi:10.3390/toxins7082845)
Supplement: Supplementary file 1 [file toxins-07-02845-s001.pdf]

Supplementary Information

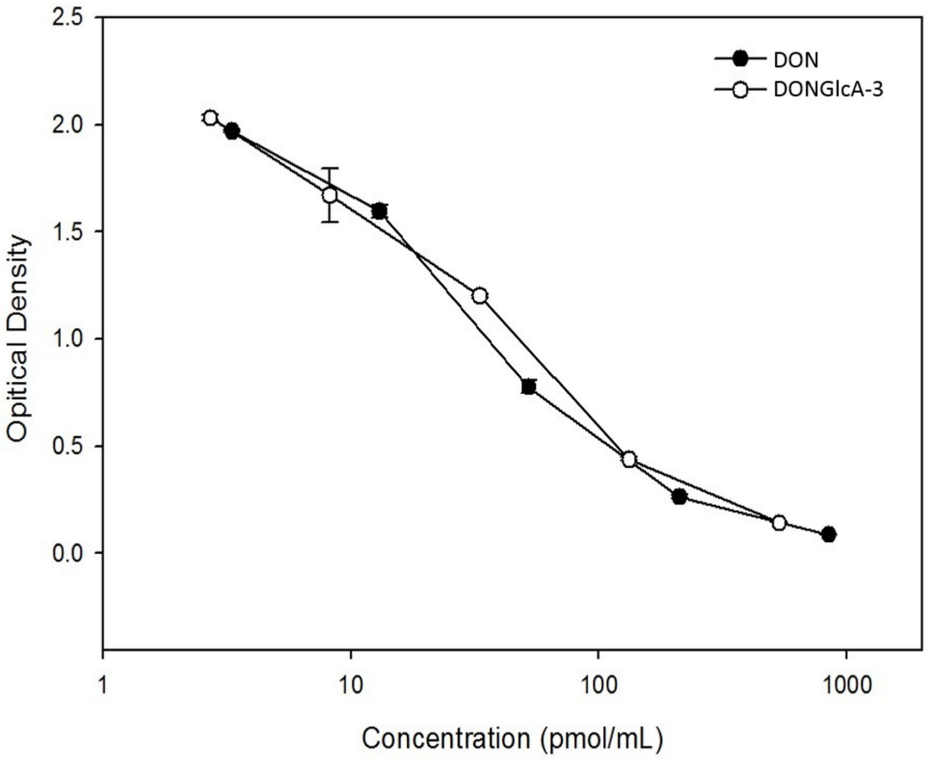

Figure S1. DONGlcA-3 is completely cross-reactive with DON Veratox high sensitivity (hs) ELISA. The DON Veratox hs ELISA is a direct competitive ELISA using Four Parameter Logistic curve-fitting analysis. The *x*-axis is logistic and the y-axis is linear. Data are mean ± SEM (*n* = 2 rep). DONGlcA-3 is the major metabolite formed in rat liver. Cross reactivity with other potential glucuronides was not determined. Since we did not measure ratio of active *vs*. conjugated DON, results in manuscript are reported as DON equivalents.

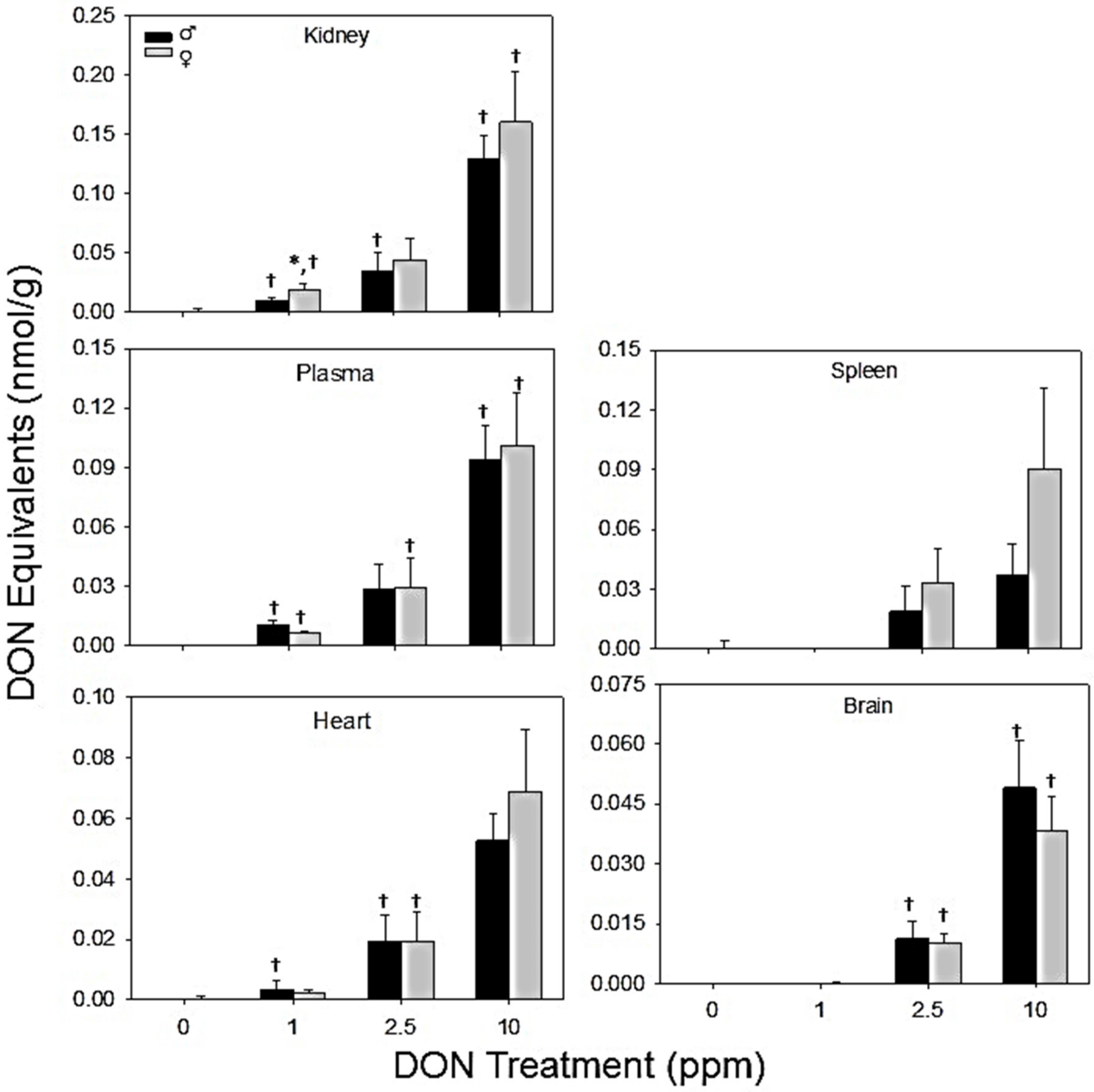

Figure S2. Organ DON equivalents increased dose dependently after dietary DON exposure in mice. Data are mean ± SEM (n = 6/gp). Asterisk indicates statistical significance from male on same treatment diet and dagger indicates significance from preceding treatment dose within sex ($p < 0.05$).
